# Effects of steam pretreatment and co-production with ethanol on the energy efficiency and process economics of combined biogas, heat and electricity production from industrial hemp

**DOI:** 10.1186/1754-6834-6-56

**Published:** 2013-04-22

**Authors:** Zsolt Barta, Emma Kreuger, Lovisa Björnsson

**Affiliations:** 1 Department of Applied Biotechnology and Food Science, Budapest University of Technology and Economics, Szt. Gellérttér 4, Budapest, H-1111, Hungary; 2 Biotechnology, Lund University, P.O. Box 124, Lund, SE-221 00, Sweden; 3 Environmental and Energy Systems Studies, Lund University, P.O. Box 118, Lund, SE-221 00, Sweden

**Keywords:** *Cannabis sativa* L., Energy crop, Biofuel, Bioenergy, Economy, Methane, Process design, Simulation, Modelling

## Abstract

**Background:**

The study presented here has used the commercial flow sheeting program Aspen Plus™ to evaluate techno-economic aspects of large-scale hemp-based processes for producing transportation fuels. The co-production of biogas, district heat and power from chopped and steam-pretreated hemp, and the co-production of ethanol, biogas, heat and power from steam-pretreated hemp were analysed. The analyses include assessments of heat demand, energy efficiency and process economics in terms of annual cash flows and minimum biogas and ethanol selling prices (MBSP and MESP).

**Results:**

Producing biogas, heat and power from chopped hemp has the highest overall energy efficiency, 84% of the theoretical maximum (based on lower heating values), providing that the maximum capacity of district heat is delivered. The combined production of ethanol, biogas, heat and power has the highest energy efficiency (49%) if district heat is not produced. Neither the inclusion of steam pretreatment nor co-production with ethanol has a large impact on the MBSP. Ethanol is more expensive to produce than biogas is, but this is compensated for by its higher market price. None of the scenarios examined are economically viable, since the MBSP (EUR 103–128 per MWh) is higher than the market price of biogas (EUR 67 per MWh). The largest contribution to the cost is the cost of feedstock. Decreasing the retention time in the biogas process for low solids streams by partly replacing continuous stirred tank reactors by high-rate bioreactors decreases the MBSP. Also, recycling part of the liquid from the effluent from anaerobic digestion decreases the MBSP. The production and prices of methane and ethanol influence the process economics more than the production and prices of electricity and district heat.

**Conclusions:**

To reduce the production cost of ethanol and biogas from biomass, the use of feedstocks that are cheaper than hemp, give higher output of ethanol and biogas, or combined production with higher value products are primarily suggested. Further, practical investigations on increased substrate concentration in biogas and ethanol production, recycling of the liquid in anaerobic digestion and separation of low solids flows into solid and a liquid fraction for improved reactor applications deserves further attention.

## Background

The European Environment Agency has identified industrial hemp (*Cannabis sativa* L.) as an energy crop whose cultivation has a lower environmental impact than crops currently used for the production of transportation fuels [1]. Biofuel production (methane or the combined production of ethanol and methane) per hectare from hemp [2-4] is higher in southern Sweden than production based on grain from wheat, triticale and rapeseed, which otherwise dominates in Sweden [5]. Hemp has a low environmental impact because it can be cultivated with relatively little nitrogen and without pesticides and it has deep roots, which improve soil structure [1,6-9]. A further benefit from increased cultivation of hemp is an increase in crop diversity. Hemp is thus an interesting energy crop that fits well with the concept of a bio-based economy in which ecosystem services other than feedstock supply are important.

The biomass yield of hemp is relatively high in southern Sweden, as it is in other parts of Europe [7,10-12]. Hemp is a lignocellulosic biomass that needs to be pretreated to achieve a high conversion degree in enzymatic hydrolysis with cellulases followed by ethanol fermentation by yeast [4]. Sipos et al. [4] optimised the SO_2_-catalysed steam pretreatment of hemp for ethanol production. The residues after ethanol production were used in a subsequent study to produce biogas (that contain methane) through anaerobic digestion (AD). The potential for producing methane by direct AD was also determined for chopped, ground and steam pretreated hemp [3]. Harsher pretreatment than chopping is not required for biogas production, but acid-catalysed steam pretreatment gives a higher production than simply chopping. Approximately half of the energy of the hemp biomass (based on the higher heating value) has practically been shown to be converted to the energy of biogas or of a combination of ethanol and biogas after steam pretreatment [3]. However, the energy requirements and the economic performance of the conversion processes have not been studied.

Energy balances and economic evaluations for the conversion of biomass to biofuels by the AD of crops are generally calculated for relatively small plants with capacities of less than 10,000 tonnes dry matter (DM) per year [5,13,14]. Walla and Schneeberger [13] have shown that the best economic performance for AD in Austria is obtained for an electricity plant of capacity 250 kW (around 1,500 tonnes DM/year). This is because plants up to this size benefit from direct subsidies. Ethanol production, in contrast, is generally analysed for large plants with capacities of more than 100,000 tonnes DM/year [15-19]. The energy efficiency and economic performance of ethanol production are, therefore, frequently compared with those of biogas production from plants that differ in size by a factor of 10 to 100 [5,20]. Several analyses have shown that the economic performance of biomass-based processes is better in larger plants [17,18,21]. The use of residues after ethanol production for AD has been analysed for large plants and shows promise [16-19]. However, no scientific papers that examine the co-production by AD with combined heat and power (CHP) at a large scale, without preceding ethanol production, have been found.

A techno-economic evaluation of a large-scale plant using 234,000 tonnes DM hemp per year has been carried out, based on experimental data from Sipos et al. [4] and Kreuger et al. [3]. Chopped hemp for biogas production, steam-pretreated hemp for biogas production, and steam-pretreated hemp for sequential ethanol and biogas production have been compared. The undegraded solids after biogas production were used for CHP in all scenarios. The effect of recycling liquid in the AD process, and the effect of using high-rate bioreactors (upflow anaerobic sludge blanket, or “UASB”, reactors) for the AD of diluted streams to partially replace continuous stirred tank reactors (CSTRs) have also been analysed. (CSTRs require longer retention times.) The process design of the ethanol and CHP part is similar to that described by Barta et al. [16], while a more detailed design of the AD process is presented here.

The aims of the current study were:

1. To determine whether the increase in biogas production from steam-pretreated hemp from that obtained from chopped hemp results in a better energy balance and economic result.

2. To determine whether combined ethanol and biogas production results in better energy balance and economic result than biogas production alone.

3. To determine whether it is economically feasible to produce biofuels from hemp.

The analysis is based on experimental data from ethanol and methane production [3,4], and it has been assumed that the same annual amount of hemp is processed in all scenarios investigated. However, it was necessary to make further assumptions and approximations, since data from full-scale biofuel production is not available. The energy efficiencies and prices that have been calculated are, therefore, not absolute. This should be borne in mind when comparing the results presented here with those of other studies.

## Results and discussion

### Process design of anaerobic digestion

The model predicts that the degradable components comprise 65-87% of the total mass (degradable and non-degradable) (Table 1). This ratio is highest at the feed to the UASB reactors and lowest at the feed to the CSTRs in Scenario Et-AD+. Recycling increases the mass flows of both the degradable and non-degradable components in the feed of AD (compare Scenario AD with AD-R and compare SP-AD with SP-AD-R). It decreases the flow of added N significantly, and increases the flows of added P and trace metals. Both P and the trace metals are recycled, but the increase in demand for these due to the increased flows of C and total mass mean that P and trace metals must be supplied.

**Table 1:** Details of anaerobic digestion in the various scenarios

**Scenario**		**AD **	**AD-R**	**SP-AD**	**SP-AD-R**	**Et-AD**	** Et-AD+**	
**AD system**		**CSTR**	**CSTR**	**CSTR**	**CSTR **	**CSTR**	**CSTR**	**UASB**
Degradable components fed^1^	t/h	22.6	27.2	22.6	24.3	18.0	10.1	7.9
In main stream^2^	t/h	18.7	23.3	17.5	19.2	9.3	6.2	3.1
In leaves	t/h	3.9	3.9	3.9	3.9	3.9	3.9	-
In flash stream	t/h	-	-	1.2	1.2	1.2	-	1.2
In liquid fraction after SP	t/h	-	-	-	-	3.6	-	3.6
Non-degradable components fed^4^	t/h	6.8	7.7	6.8	7.7	6.8	5.6	1.2
In main stream^2^	t/h	6.3	7.2	6.3	7.2	5.4	5.1	0.3
In leaves	t/h	0.5	0.5	0.5	0.5	0.5	0.5	-
In flash stream	t/h	-	-	0	0	0	-	0
In liquid fraction after SP	t/h	-	-	-	-	0.9	-	0.9
C flow fed^5^	t/h	13.2	16.8	13.2	14.8	10.1	4.4	5.6
N added	kg/h	370	185	370	274	67	0	227
P added	kg/h	45.1	61.0	45.1	50.9	0	14.1	0
Fe added	kg/h	17.3	21.2	14.3	16.2	30.1	30.1 ^6^	
Ni added	g/h	4.5	8.8	0	0.8	29.1	29.1 ^6^	
Co added	g/h	97	114	82	90	162	162 ^6^	
Degradation ratio^3^	-	0.53	0.48	0.68	0.65	0.66	0.65	0.49
Sludge DM in the effluent	t/h	1.29	1.42	1.55	1.60	1.23	0.67	0
Raw biogas produced	Nm^3^/h	9412	11275	10803	11456	7755	4425	3254

A summary of the scenarios is given in Figure 3.

AD: anaerobic digestion, CSTR: continuous stirred tank reactor, UASB: upflow anaerobic sludge blanket, SP: steam pretreatment, DM: dry matter

^1^ This value refers to carbohydrates, proteins, lipids, extractives, organic acids, ethanol, glycerol, enzymes, yeast, and sugar degradation products.

^2^ This figure refers to hemp stems, or steam pretreated hemp stems, or whole stillage, or thin and thick stillages, depending on the scenario. It includes also the recycled liquid fraction of the AD effluent.

^3^ The degradation ratio is defined as the difference between the mass flow of degradable components in effluent and input, divided by the mass flow of degradable components input.

^4^ Water-insoluble and water-soluble lignin, ashes and other unknown components are considered to be non-degradable.

^5^ This value includes the carbon flow of the recycled liquid fraction of the AD effluent.

^6^ Experimental data for trace metal contents are available only for the feedstock (Table 2), and thus the distribution of trace metals between CSTR and UASB is unknown. The total requirements of the two systems have therefore been assumed to be the same as those of Scenario Et-AD.

The mass flow of non-degradable components is the same in the feed as in the effluent, while 48-68% of the degradable material is broken down during AD (Table 1, in which the definition of degradable and non-degradable components is also given). The relatively low degradation degree indicates either a higher formation of sludge or a higher concentration of non-degradable compounds than assumed. It could also indicate that it is possible to optimise the pretreatment further to improve the accessibility of degradable compounds and/or decrease the formation of non-degradable or inhibiting compounds. To elucidate the reason, continuous anaerobic digestion experiments, with steam pretreated biomass, including analysis of the compounds present after anaerobic digestion is suggested in future research.

The components of the flash stream are volatile organic substances, and are considered as degradable compounds. The flows of C, degradable components and non-degradable components obtained after steam pretreatment and fed to AD (in Scenario SP-AD), without the use of recycling, are equal to those fed directly to AD in Scenario AD, since the solid material that is lost during pretreatment (in the flash stream) is recovered by feeding the flash stream to AD. The amounts of macronutrients added are the same since the flows of C are the same, and since N and P are entirely recovered after pretreatment, either in the whole slurry or in the flash stream. However, the addition of trace metals is based on the concentrations in the input flow, and the feed of AD after water dilution differs in Scenario SP-AD (168 tonnes/h) from that in Scenario AD (198 tonnes/h). Less trace metals are added after steam pretreatment as a consequence (Table 1). The mass flow of feed in Scenario AD is greater than that in Scenario SP-AD, since the dilution of the feed is based on the DM concentration at the end of AD (10% in both scenarios) and the volatile compounds that form during steam pretreatment are not considered as DM. This means that less DM is fed to AD in Scenario SP-AD than in Scenario AD (part of the feedstock DM is lost during pretreatment), and less dilution water is therefore used. The Ni demand in AD carried out after pretreatment is decreased to such extent that the Ni present in hemp is sufficient, hence extra Ni does not need to be added (Table 1).

The amounts of N and P that must be added are lowest in Scenario Et-AD, because of the molasses and macronutrients that are added in the yeast cultivation and SSF steps. The overall addition of macronutrients is greater in Scenario Et-AD + than in Scenario Et-AD, due to the distribution of macronutrients between the UASB and CSTR systems. Experimental data for levels of trace metals are available only for the feedstock (Table 2), and the distribution of trace metals between CSTR and UASB is not known. It has, therefore, been assumed that the total demands of the two systems are the same as those in Scenario Et-AD. In Scenarios AD-R and SP-AD-R, 153 and 62 tonnes of liquid fraction per hour, respectively, are recycled. Less degradable material is fed, less raw biogas and less sludge are produced in Scenario SP-AD-R than in Scenario AD-R, as the recycled liquid flows are lower (Table 1). The effluent from the UASB reactors does not contain sludge as sludge granules are retained in the reactors.

**Table 2:** Composition, macronutrient (N, P) contents and trace metal (Fe, Ni, Co) contents of hemp stems and leaves used in the model

		**Stems**	**Leaves**
Glucan	% of DM	43.6^1^	21.4^2^
Mannan	% of DM	1.9^1^	1.8^2^
Galactan	% of DM	2.0^1^	3.4^2^
Xylan	% of DM	10.5^1^	2.2^2^
Arabinan	% of DM	0.6^1^	2.3^2^
Acetate	% of DM	2.3^1^	-^3^
Lignin	% of DM	21.5^1^	-^3^
Proteins	% of DM	3.1	21.9^6^
Lipids	% of DM	1.8	-^3^
Volatile extractives	% of DM	1.8^1^	-^3^
Non-volatile extractives	% of DM	7.2^1^	38.3^2^
Others	% of DM	3.6^1^	11.4^2^
Total N	g/kg DM	5.0	35.0
P	g/kg DM	2.7	5.0
Fe	mg/kg DM	86.7^4^	
Ni	mg/kg DM	1.2^4^	
Co	mg/kg DM	0.1^4^	

DM: dry matter.

^1^ Based on Sipos et al. [4].

^2^ Based on Kreuger et al. [2].

^3^ The value for leaves is not available, a value of zero has therefore been used in the model.

^4^ Determined for the whole plant.

### Overall heat demand and energy output

The overall heat duty can be decreased by means of heat integration to 72, 61 and 30% in Scenario AD, Scenario SP-AD and Scenario Et-AD, respectively (calculated from Table 3). It can be concluded that the more high-temperature steps (steam pretreatment, distillation) that the process contains, the more important will be the role played by heat integration. It must be pointed out that these structures of heat integration are near-optimal in terms of both capital cost and heat demand. The increase in capital cost required at higher degrees of integration outweighs the reduction in cost due to a lower heat demand.

**Table 3:** Thermal and electrical data and energy flows of products in the various scenarios, expressed in MW

	**AD **	**AD-R**	**SP-AD**	**SP-AD-R**	**Et-AD**	**Et-AD+**
Heat duty without HI	12.4	10.8	30.3	31.1	73.1	70.0
Heat duty after HI	8.9	6.6	18.4	18.4	21.6	21.3
23 bar steam injected to SP	-	-	13.7	13.7	13.7	13.7
4 bar steam injected to SP	-	-	4.7	4.7	4.7	4.7
4 bar steam, indirect heating	4.7	5.6	-	-	3.2	2.9
90°C hot water	4.2	1.0	-	-	-	-
District heat produced^1^	52.2	56.9	39.9	40.7	22.9	17.9
From FGC	21.2	22.3	16.3	16.6	12.3	9.3
From the process^2^	-	-	11.0	11.0	7.0	7.0
From steam cycle	35.2	35.6	12.6	13.2	3.6	1.6
Electricity generated	16.2	16.6	9.3	9.6	6.5	5.5
Electricity sold(+)/purchased(-)	10.9	10.5	4.4	4.2	-1.2	0.3
Biogas (based on LHV)	53.1	63.6	65.9	69.8	50.1	50.1
Ethanol (based on LHV)	-	-	-	-	34.1	34.1

A summary of the scenarios is given in Figure 3. The energy flow of the feedstock is 155.2 MW.

HI: heat integration, SP: steam pretreatment, FGC: flue gas condensation, LHV: lower heating value.

^1^ Reduced by the duty required to heat water to 90°C for heating the process. This is the maximum capacity: the average annual capacity can be calculated by applying a factor of 0.56, which corresponds to the following assumptions: heat is delivered to the district heating system during a period of time equivalent to 4,500 hours of the maximum annual capacity. Cooling water is used during the remaining 3,500 hours to remove the heat [23].

^2^ Excluding combined heat and power production.

In Scenario AD, 47% of the overall heat duty can be covered by using hot water at 90°C obtained during the production of district heat. Hot water usage and the overall heat duty are lower in Scenario AD-R (Table 3), since the recycled liquid stream is at the temperature of AD (37°C), and does not require preheating before input to the AD step.

In Scenarios SP-AD and SP-AD-R, only steam injected directly into the steam pretreatment (at 4 and 23 bar) is required as heating medium, and this is obtained from the CHP plant (Table 3). Thus, the heat losses in AD and the heat demands of biogas upgrading and preheating of the make-up water of the CHP plant can be supplied by heat that is available in the process. In scenarios Et-AD and Et-AD+, 14-15% of the overall heat duty is covered by the steam at 4 bar that is used in indirect heating.

District heat is produced in Scenarios AD and AD-R from flue gas condensation and the steam cycle, while in other scenarios (SP, SP-R, Et-AD and Et-AD+) significant amounts of heat can be recovered as district heat from the process itself (Table 3). Recycling increases the amount of electricity generated (compare Scenario AD-R with AD and compare SP-AD-R with SP-AD in Table 3), as the energy flow to the CHP plant is higher (data not shown). Similarly, less power is generated in Scenario Et-AD + than in Et-AD (Table 3), since only the solid fraction of the CSTR effluent is incinerated (the effluent from the UASB reactors is passed to wastewater treatment). Electricity is a co-product in all scenarios except Scenario Et-AD. The power requirement of Scenario Et-AD is 48% higher than that of Scenario Et-AD + (Table 3). The difference is primarily due to the higher power consumption of pumps and agitators in the AD system. The rates of production of ethanol (5,800 L/h) and of upgraded biogas (5,024 Nm^3^/h) are assumed to be equal in Scenarios Et-AD and Et-AD+, as experimental data for the continuous systems are not available.

### Energy efficiency

**Figure 1:**
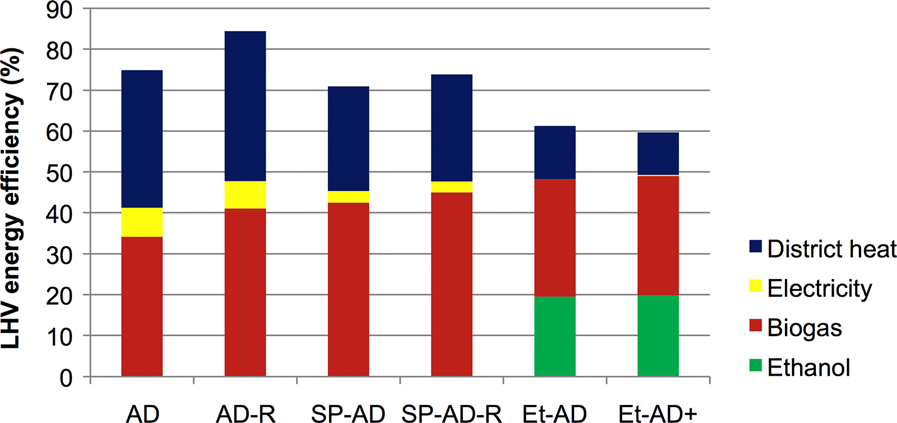
**Energy efficiency.** Overall energy efficiency at maximum district heat delivery, based on lower heating values (LHV), expressed as percentage of the input. A summary of the scenarios is given in Figure 3.

The highest overall energy efficiency (84% of the theoretical maximum) is obtained in Scenario AD-R (Figure 1) providing that the maximum capacity of district heat is delivered. District heat delivery is zero during the summer, however, which means that the overall energy efficiency decreases to 41-49% in the scenarios investigated. The highest efficiency without district heating is obtained in Scenario Et-AD+. The efficiency of this scenario is lower than that of the corresponding scenario (Scenario B) of Barta et al. [16]. This is due to several effects: the composition of the feedstock differs, as do the process yields. Furthermore, the WIS concentration in SSF is lower (7.5 instead of 10%). Barta et al. [16] assumed also that the aerobic sludge from wastewater treatment is incinerated in the CHP plant, which is not the case in the present study. Although energy efficiency is calculated based on LHV in both cases, the present study uses the LHV of dry feedstock, while Barta et al. [16] considered the LHV of wet feedstock. Processes with higher heat demands have lower overall energy efficiency, since less energy remains in the form of products (Table 3 and Figure 1).

The energy demand (excluding the energy required for feedstock production and transport) of Scenarios AD, SP-AD and Et-AD + are 14%, 20% and 22% (in heat equivalent) of the energy in the feedstock, respectively (based on LHV), Table 3. In Scenario AD, the complete energy demand is covered by CHP produced from the solid residues after AD. In smaller-scale methane production, where CHP of the solid residue is normally lacking, part of the biogas or other energy sources is needed to cover the energy demand of the process [5]. Svahn [24] has shown that the use of insulated AD reactors in Scenario AD decreases heat losses by 88%, and in this case the total energy demand (heat and power in heat equivalent) is 13% of the energy in the feedstock, based on LHV. The energy required by cooling processes in the other scenarios is much higher, and it can be reduced by transferring heat to non-insulated AD reactors to cover the heat losses from these. Recycling part of the liquid (as is done in Scenario AD-R) does not influence the total energy demand (in heat equivalent) of the process, but slightly more electricity and less heat is needed (Table 3).

### Capital investment

The direct costs of pretreatment, SSF, AD and CHP are significant (Table 4). The introduction of recycling increases the direct costs of AD since the higher amount of DM that is processed during AD requires reactors of larger volumes. Similarly, Scenario AD has higher direct costs than Scenario SP-AD due to its higher DM flow. The separate biogas production in CSTR and UASB systems significantly reduces the direct costs of AD, as the shorter retention time in the UASB allows reactors of lower volumes to be used. Combined production of ethanol and biogas is more capital-intensive than direct AD and AD with preceding steam pretreatment. Using separate CSTR and UASB systems during combined production (Scenario Et-AD+) reduces of the capital investment by 17% from that of a system in which all of the stillage is digested in CSTR reactors (Scenario Et-AD) (Table 4). However, the capital investment of UASB reactors may be underestimated in Table 4, as it is based on the size of the tank and does not include the cost of settlers and any extra licensing fees required to use designs that are protected by patents. Scenario SP-AD does contain an extra process step, steam pretreatment, that Scenario AD does not have, but even so, the total capital cost of Scenario SP-AD is lower than that of Scenario AD (Table 4), mainly due to the capital costs of AD and CHP being lower.

**Table 4:** Breakdown of the total capital investment cost in million Swedish Kronor

	**AD **	**AD-R**	**SP-AD**	**SP-AD-R**	**Et-AD**	**Et-AD+**
Feedstock handling	9	9	9	9	9	9
Pretreatment	-	-	115	115	115	115
YC & SSF	-	-	-	-	123	123
Distillation	-	-	-	-	30	30
Anaerobic digestion	228	283	174	215	356	141
Separation	37	42	30	30	59	79
Biogas upgrading	54	60	59	61	48	48
Combined heat and power production^1^	154	157	124	125	104	97
Storage	3	3	9	9	25	25
Heat exchanger network	3	3	8	9	34	37
Total direct costs	487	558	529	574	905	705
Total indirect costs	660	702	521	551	855	745
Fixed capital^2^	1148	1259	1050	1125	1760	1450
Working capital	12	12	13	13	32	32
Total capital investment^3^	1160	1272	1063	1138	1792	1482

A summary of the scenarios is given in Figure 3. 1 EUR ≈ 8.9 SEK.

YC: yeast cultivation, SSF: simultaneous saccharification and fermentation.

^1^ Includes the flue gas condenser.

^2^ Sum of direct and indirect costs.

^3^ Sum of fixed capital and working capital.

### Annual cash flow

All scenarios run at a deficit, with income lower than the costs. The deficits for Scenario SP-AD and Scenarios Et-AD and Et-AD + differ from that of Scenario AD by less than 10% (Table 5). These processes could be made economically viable by decreasing total costs by 28-36%. The cost of feedstock is the greatest contribution to the costs, followed by the capital cost (Table 5). Ljunggren and Zacchi [25] analysed AD of potato steam peels using enzymatic liquefaction and saccharification as pretreatment, and producing upgraded biogas. They found that the costs of capital and nutrients were the main contributors to the production cost and the process was not feasible economically mainly due to the small scale (2 MW upgraded biogas was produced at a production cost of approximately 2000 SEK/MWh). The scale of the AD plant is an important issue in the context of economics. Brown et al. [26] and Yiridoe et al. [27] found that without incentive schemes and considering nonmarket cobenefits, on-farm biogas energy production was not economical under a farm size of 600-sow in Nova Scotia, Canada. The cost-effective size range of AD systems without subsidies was shown to be a digester volume of 800 m^3^ and up, under the assumptions set for Greek pig farming [28]. Higham [29] investigated the economics of two generic AD plants based on real data of European plants processing agricultural wastes: a farm scale digester and a centralised digester with 25 kW and 1 MW electrical export capacity, respectively. The base cases showed that none of the generic plants were economically attractive, however, applying assumptions of achievable improvements the scenarios became economically feasible.

**Table 5:** Annual cash flows in million Swedish Kronor

	**AD **	**AD-R**	**SP-AD**	**SP-AD-R**	**Et-AD**	**Et-AD+**
Costs						
Feedstock	-381	-381	-381	-381	-381	-381
Capital	-127	-139	-116	-124	-195	-161
Chemicals	-22	-13	-35	-30	-42	-51
Enzymes	-	-	-	-	-48	-48
Utilities	-1	-1	-1	-1	-6	-1
WWT	-19	-5	-16	-10	-41	-41
Others^1^	-21	-21	-20	-21	-23	-22
Total cost	-570	-560	-570	-568	-736	-705
Incomes						
Ethanol	-	-	-	-	255	255
Biogas	255	305	316	335	241	241
Electricity	48	46	19	18	0	1
District heat	66	72	50	51	29	23
Total income	368	423	386	405	525	520
Deficit	202	137	184	163	211	185

A summary of the scenarios is given in Figure 3. 1 EUR ≈ 8.9 SEK.

WWT: wastewater treatment.

^1^ ‘Others’ includes maintenance, insurance and labour.

The difference in the cost of chemicals between Scenarios SP-AD and AD is primarily due to the cost of SO_2_. The costs of macronutrients are equal, and the costs of trace metals are only 2% and 3% of the total chemical costs in these scenarios. The costs of chemicals in Scenario Et-AD + are 21%, higher than those in Scenario Et-AD, since having separate CSTR and UASB systems leads to a higher requirement for macronutrients (Table 1). This is a consequence of the unequal distribution of organic materials and macronutrients at separation. The enzyme costs in combined scenarios (Et-AD and Et-AD+) are 6-7% of the total costs. The costs for utilities are negligible, since they only contain the cost of the cooling water and the process water used as make-up water in the CHP plant to produce the steam that is injected directly into the steam pretreatment stage, and the cost of electricity (in Scenario Et-AD). Process water is not required for dilution before AD and SSF: this water is supplied in the model from water passed through the on-site wastewater treatment. Recycling is beneficial in both cases in which it is applied, primarily due to the increase in volume of methane produced that results from using it. However, the increase in methane production is not necessary to obtain the same or a lower deficit as that obtained without recycling (Table 5). Scenarios AD and SP-AD have the same total cost. The two major sources of income are the biogas and the ethanol, while the electricity and the district heat contribute to income to a lesser extent.

The economic consequences of storing the liquid fraction of the AD effluent and its subsequent use as fertiliser have been investigated. The liquid in this case is stored for 11 months, then it is transported to agricultural land and spread. The cost of spreading was assumed to be equal to the income from selling the liquid fraction as fertiliser (data are not available for either of these). The least liquid is released to wastewater treatment in Scenario AD-R (Table 5), and a storage capacity of 280 kilotonnes liquid/year is required. The N, P, Fe, Ni and Co contents of the stored liquid in this scenario were estimated to be 2340, 131, 5, 0.03 and 0.04 mg/L, respectively. The annual capital cost increases by 47 MSEK, the cost of wastewater treatment (5 MSEK) is eliminated, and the income from the sale of electricity decreases by 1 MSEK due to the power consumed by the electrical systems of the carbon steel storage tanks. Other cost elements remain the same, and thus storing the liquid instead of wastewater treatment increases the total cost (to 602 MSEK) and decreases the total income (to 422 MSEK).

### Minimum selling prices and sensitivity to market prices

The MBSP and MESP that were calculated (Table 6) are higher than the assumed market prices of biogas and ethanol (Table 7), respectively. Barta et al. [16] analysed large-scale spruce-based ethanol processes, in which upgraded biogas is also produced. The ethanol production cost (equivalent to MESP) was much lower (4.00 SEK/L) than those obtained here, mainly due to the lower price of feedstock. Ekman et al. [30] presents an analysis for large-scale combined ethanol and biogas production. Profit is reached under Swedish conditions based on cereal straw as substrate, which is also considerably cheaper than hemp. The present study shows that the recycling can improve the process economics according to the assumptions applied in the model. Steam pretreatment before AD without recycling (Scenario SP-AD) is economically more favourable than direct AD without recycling (Scenario AD), but the MBSP of steam pretreatment with recycling (Scenario SP-AD-R) is higher than that of direct AD with recycling (Scenario AD-R). It can be concluded that the positive economic effect of steam pretreatment before AD depends largely on the increase of methane production caused by steam pretreatment. Separate CSTR and UASB systems during combined biofuel production are more favourable in terms of both the MBSP and MESP (Scenario Et AD+ in relation to Et AD, Table 6). The MBSP of Scenario Et-AD + is slightly lower than that of Scenario AD, while it is higher than that of Scenario SP-AD. The latter scenario is economically the most favourable among the cases without recycling, while Scenario AD-R has the lowest MBSP of all scenarios (Table 6). The feedstock prices at the break-even point are 45-64% of the assumed feedstock price (Table 6).

**Table 6:** Minimum biogas and ethanol selling prices (MBSP and MESP, respectively), feedstock price at the break-even point and sensitivity analysis of MBSP

	**AD **	**AD-R**	**SP-AD**	**SP-AD-R**	**Et-AD**	**Et-AD+**
MBSP (SEK/MWh)	1076	869	949	893	1123	1059
MESP (SEK/L)	-	-	-	-	10.02	9.47
Feedstock price at break-even point (SEK/dry t)	776	1058	855	943	742	853
MBSP (SEK/MWh) if prices change						
Feedstock price -50%	627	494	587	551	648	584
Feedstock price +50%	1525	1243	1310	1234	1598	1534
Ethanol price -50%	1076	869	949	893	1441	1377
Ethanol price +50%	1076	869	949	893	805	741
Electricity price -50%	1132	914	967	909	1123	1061
Electricity price +50%	1019	824	930	876	1123	1057
District heat price -50%	1153	939	996	939	1159	1087
District heat price +50%	998	798	901	847	1087	1031

A summary of the scenarios is given in Figure 3. 1 EUR ≈ 8.9 SEK.

SEK: Swedish Kronor.

**Table 7:** Prices associated with operational costs and products

	**Price (SEK)**		**Unit**	**Reference**
Feedstock	1.62		kg DM	-
Chemicals				
Sulphur dioxide	1.5		kg	[15]
Antifoam	20		kg	[15]
(NH_4_)H_2_PO_4_	1.4		kg	[31]
MgSO_4_	4.4		kg	[15]
Molasses	1.0		kg	[15]
Urea	3.0		kg	[31]
FeSO_4_.H_2_O	1.1		kg	[32]
NiCl_2_	41		kg	[32]
CoSO_4_.7H_2_O	67		kg	[32]
Cellulase enzymes	28.5		MFPU	[22]
Utilities				
Electricity (cost)	450		MWh	[23]
Cooling water	0.14		m^3^	[15]
Process water	1.40		m^3^	[15]
Products				
Ethanol	5.5		L	[23]
Biogas	600		MWh	[16]
Electricity, spot price	350		MWh	[23]
Electricity certificate	200		MWh	[23]
District heating	280		MWh	[23]
Cost of wastewater treatment	160.5		m^3^	Estimated from [16]

SEK: Swedish Kronor (1 EUR ≈ 8.9 SEK), DM: dry matter, MFPU: million filter-paper units.

**Figure 2:**
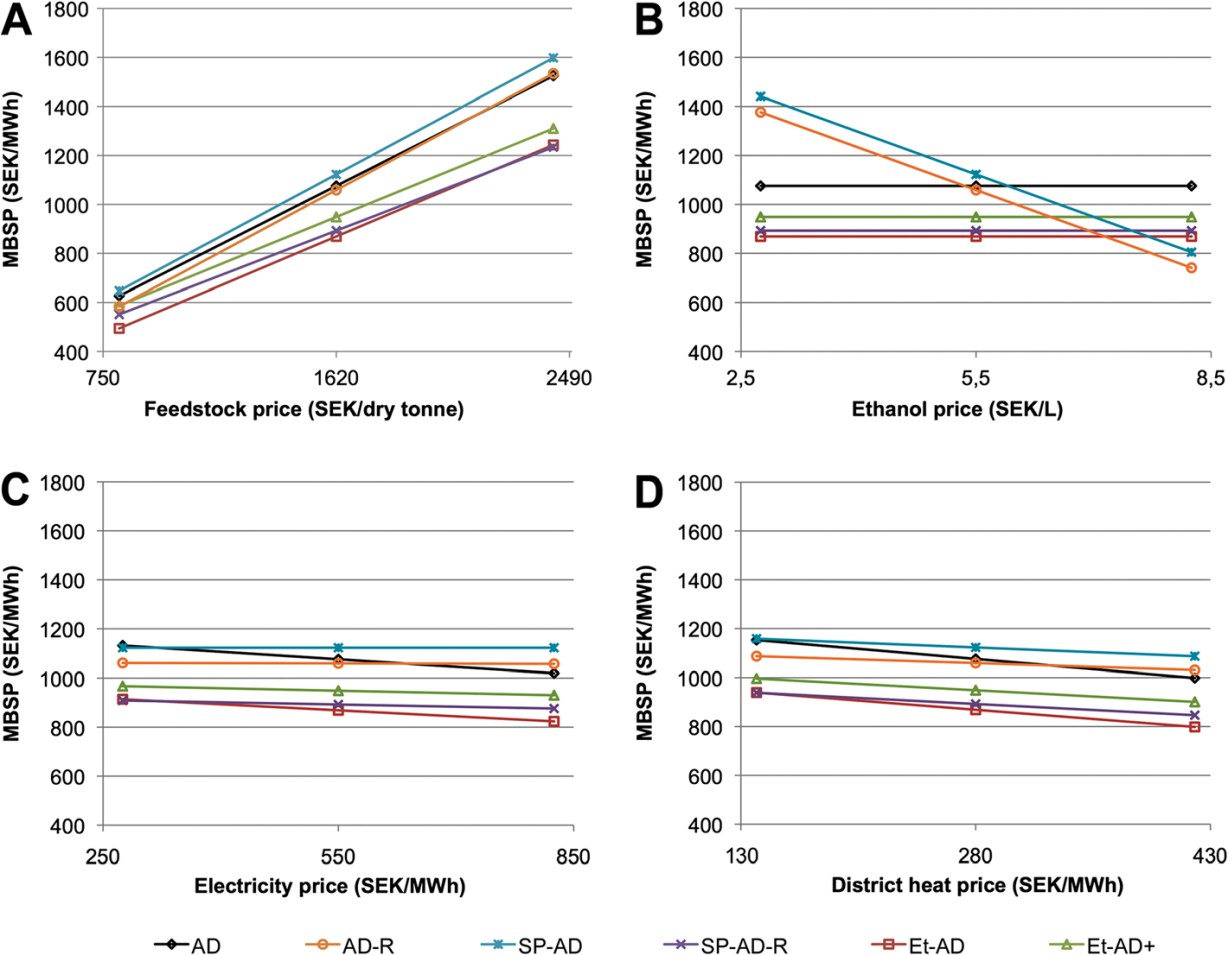
**Minimum biogas selling price.** Minimum biogas selling price (MBSP, in Swedish Kronor (SEK)/MWh) as a function of **(A)** feedstock price, **(B)** ethanol price, **(C)** electricity price, and **(D)** district heat price. 1 EUR ≈ 8.9 SEK. A summary of the scenarios is given in Figure 3.

The sensitivity of the MBSP to changes in the prices of feedstock and of products has been examined by changing one price at a time from -50% to +50% (Figure 2 and Table 6). If the feedstock price decreases by 50%, the MBSP in most scenarios (AD-R, SP-AD, SP-AD-R, Et-AD+) falls below the assumed market price of biogas (600 SEK/MWh), and these scenarios become economically feasible (Figure 2A). If the market price of ethanol increases by 75%, Scenario Et-AD + would become economically viable (Figure 2B). Changes in the prices of electricity (Figure 2C) and district heat (Figure 2D) have little influence on the process economics. Further, it is not likely that these prices will increase to such an extent that the MBSP of any of the scenarios falls below the market price of biogas.

## Conclusions

Energy output in the form of biogas, ethanol, heat and electricity lies between 60 and 84% of the energy input in the processes that have been analysed. None of these processes is economically viable. Steam pretreatment of chopped hemp before biogas production changes the deficit by less than 10% compared to direct biogas production from chopped hemp followed by CHP production from the solid residues. Similarly, the deficit of a combined process to produce both ethanol and biogas is also less than 10% lower than that of producing biogas alone. The cost of feedstock is the largest contribution to costs, but it must be stressed that the cost of producing ensiled hemp is uncertain, since hemp is not currently produced for this purpose. Nevertheless, the feedstock price prevents the processes becoming feasible, and it must be reduced to approximately half to make hemp a feasible feedstock, else a cheaper feedstock needs to be chosen. Alternatively, the total production costs must be reduced by around one third in order to achieve economical viability. Improving the processes by, for example, increasing the concentration of solids in the SSF and AD, or by decreasing the retention times for these process steps, can reduce significantly the capital investment required by these process steps. Furthermore, increasing the concentration of solids also has a positive effect on the downstream processes, such as distillation and wastewater treatment. Recycling of liquid in AD of chopped hemp has a positive influence on the MBSP, especially if the methane production can be increased in this way. It would, therefore, be interesting to investigate the influence of recycling in practice. Further, the separation of low solids streams to a solid and liquid fraction for improved reactor application was shown to improve the process economy and deserves to be practically verified.

The production and prices of methane and ethanol have larger influences on the process economics than the production and prices of electricity and district heat. The yields and prices of methane and ethanol have a larger effect also than the choice of process combination and configuration. It would, therefore, be interesting to analyse feedstocks that give higher production of biogas and ethanol.

It may be possible to improve the economic feasibility of biofuel production by combined production of value-added products, such as hemp fibres. Barta et al. [33] have shown that the hemp hurds that remain after mechanical separation of bast fibres can be used for biofuel production.

## Methods

### General process data and feedstock composition

The processes that have been modelled are referred to as “scenarios” below. It has been assumed that they are implemented in the County of Scania, Sweden, and convert 234,000 tonnes of hemp DM annually (200,000 tonnes of stems DM with the corresponding weight of leaves), and the time of operation is 8000 hours per year. Feedstock composition and experimental yields from steam pretreatment, simultaneous saccharification and fermentation (SSF), and AD have been reported in recent publications [2-4]. The study described here includes some additional analyses. Nutrients (C, N, P, K, Mg, Ca, Na, Si, Fe, Al, B, Cu, Zn, Mn) of stems, leaves, steam pretreated stems, solid and liquid fraction of steam pretreated stems and the SSF residue, by the commercial lab LMI AB according to previously described methods [34]. Protein content was calculated as 6.25 times the mass nitrogen [35]. Data on fat content was based on another September harvested hemp sample of the same cultivar, determined as raw fat [35] by the commercial lab Eurofins Sweden AB. The methane production from AD is based on biochemical methane potential batch tests [3], which have been assumed to be representative for continuous digestion. All analyses refer to the hemp cultivar Futura 75, cultivated at 55° north, 13° east, and harvested in September. Table 2 summarises the feedstock composition.

### Scenario AD – Direct anaerobic digestion

**Figure 3:**
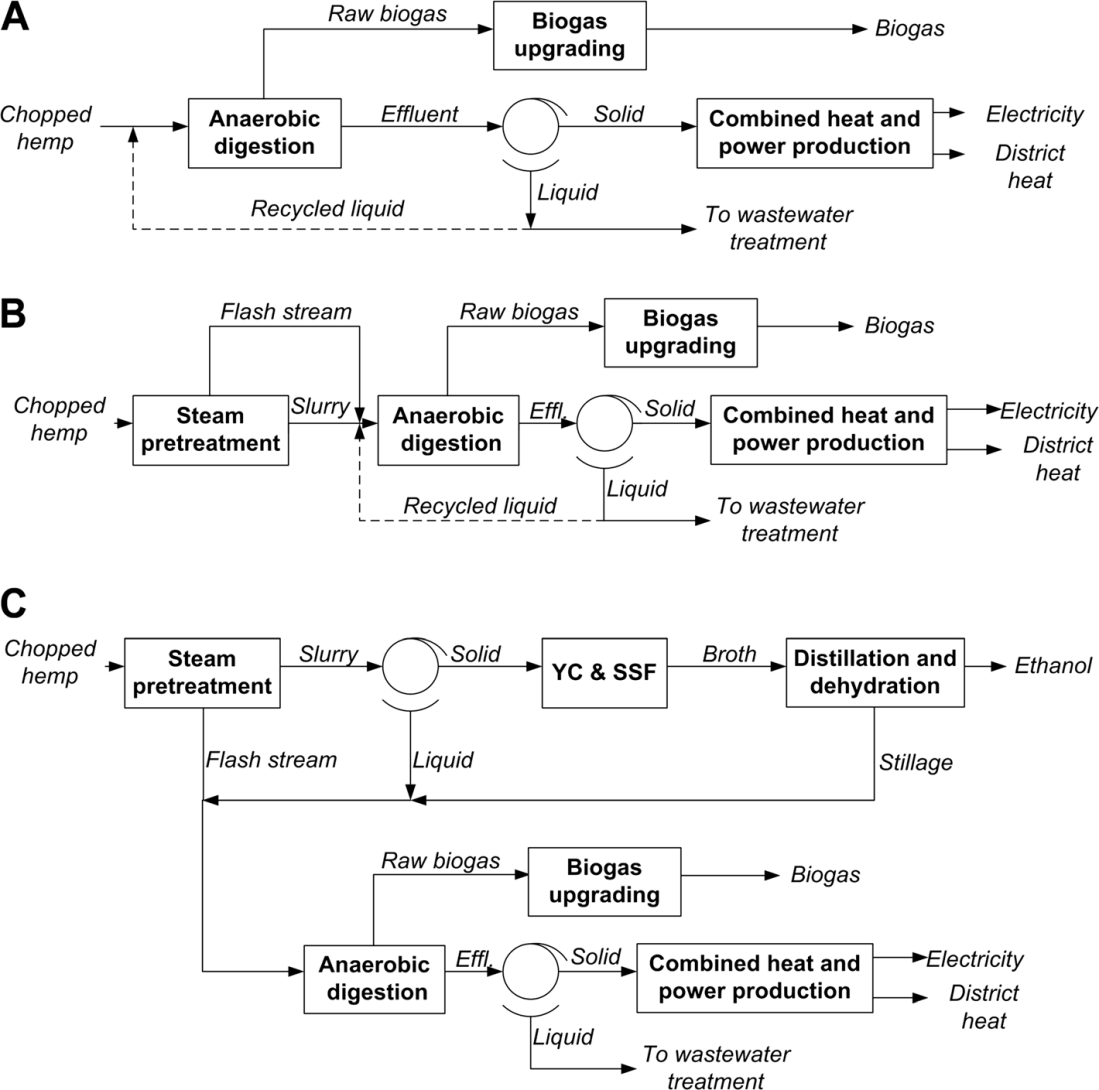
**Process schemes.** Process schemes of: **A)** direct AD (Scenarios AD and AD-R), **B)** steam pretreatment prior to AD (Scenarios SP-AD and SP-AD-R), and **C)** combined ethanol and biogas production (Scenarios Et-AD and Et-AD+). The dashed lines in Figures A and B show flows that are used in Scenarios AD-R and SP-AD-R. Water (not shown) is used for dilution in Scenarios AD and SP-AD. Effl.: effluent, YC & SSF: yeast cultivation and simultaneous saccharification and fermentation.

Scenario AD involves AD of chopped hemp in CSTR systems at 37°C, with a total average hydraulic retention time of 30 days. Chopped hemp with a DM content of 30% [2] is diluted with water before being fed to the first four reactors. The DM concentration at the end of AD (in the effluent of the final reactor) is 10% (Figure 3A). The CSTR system is composed of identical reactors, each with a volume of 10,000 m^3^ (with a ratio of working volume to total volume of 0.85) and a height-to-diameter ratio of 1.5. The reactors are arranged in blocks, each one of which consists of five reactors. Four reactors are connected in parallel, and the effluents from these are mixed and fed to the fifth reactor. The retention time in the first four reactors is 24 days, while it is 6 days in the fifth reactor. The organic loading rate for all reactors is 5 kg DM/(m^3^d). Serial digestion was chosen as the basis of the model, since it can give a higher methane production per kg feedstock and per m^3^ reactor for a given retention time than one-step digestion [36]. By taking into account the N and P present in the feedstock, urea (CO(NH_2_)_2_) and ammonium phosphate ((NH_4_)H_2_PO_4_) are added in this scenario to adjust the C/N and C/P ratios during AD to 20 and 100, respectively [37,38]. Beside the metal contents of the hemp, trace metals in the form of FeSO_4_.H_2_O, NiCl_2_ and CoSO_4_.7H_2_O are added to achieve concentrations of 100, 0.2 and 0.5 mg/L, respectively, [39]. The power required for feeding the raw material is 1.9 kWh/tonne wet hemp (Personal communication, Läckeby Water Group AB) and that required for stirring is 10 kWh/m^3^ slurry [40]. The digesters are not insulated and the overall heat loss is assumed to be 170 W/m^2^ (through the foundation and through the wall below the liquid level) [24].

The model of AD was based on the following stoichiometric reactions: hydrolysis of polysaccharides into monomeric sugars, sludge (microbial biomass) formation and biogas production. Sludge and biogas are produced from the following degradable compounds (where the assumed degradation factors are given in parentheses): sugars, proteins, lipids, acetic acid (1.00) and extractives (0.25). Hence, part of the extractives, together with the unhydrolysed polysaccharides, lignin and ‘Others’, was considered to be non-degradable in terms of sludge formation and biogas production. Ten percent of the amount of each compound that is degraded was assumed to form sludge, while 90% is converted into biogas. Equal hydrolysis conversion factors were assumed for all polysaccharides, and it was possible to calculate these from the experimental methane production.

The entire amount of biogas is upgraded by applying the amine absorption technology known as “CApure” (Personal communication, Läckeby Water Group AB) (Figure 3A). This technology guarantees a methane recovery greater than 99.9% and a methane purity of 99.3%. The upgraded biogas that is produced was assumed to be injected into the main stem of the Swedish natural gas grid, and it is therefore necessary to increase its pressure to 28 bar. The heat and power required to upgrade are 0.5 and 0.17 kWh/Nm^3^ raw biogas, respectively. The heat must be supplied at least as low-pressure steam (>3.5 bar), and 75% of the heat required can subsequently be recovered by heating water to 60°C.

The effluent from the AD is separated by filter pressing, to give a solid fraction with a DM concentration of 40% and a water-insoluble solid (WIS) retention of 99% (Figure 3A). The liquid fraction is subjected to wastewater treatment, the effluent from which is clean water and can be used for dilution in the process. Wastewater treatment is not included in the process model, but the estimated total cost of it is included in the economic evaluation. The solid fraction is incinerated on site to generate steam and electricity. The CHP step has been described elsewhere [16]. District heat is produced by using the heat of flue-gas condensation and the heat available in the steam cycle. A detailed description of the Swedish district heating system included in the model has been given by Sassner and Zacchi [23].

### Scenario SP-AD – Steam pretreatment prior to anaerobic digestion

Sassner et al. [15] have described the process model for steam pretreatment. Steam pretreatment of chopped hemp stems is performed at 210°C for 5 min, with the addition of 2% SO_2_ as catalyst [4]. (The conversion factors for some of the reactions are: glucan to glucose 0.002, xylan to xylose 0.084, xylan to furfural 0.221, water-insoluble lignin to water-soluble lignin 0.100). The steam pretreated slurry is subjected to AD, together with condensed flash vapours from the pretreatment and chopped leaves (Figure 3B), where AD is performed in the same way as in Scenario AD (the degradation factor of furfural and hydroxymethylfurfural is assumed to be 0.9, while soluble lignin is considered to be non-degradable; the degradation factor of other components is given at Scenario AD). The methane production from AD in the model was assumed to be higher than the value obtained from experimental data [3], since organic compounds in the condensed flash vapours are also degraded. This was not the case in the experimental situation, since flash vapours were not condensed [3]. Oligosaccharides are released during pretreatment, and these are entirely converted into biogas and sludge during AD. The biogas upgrading and the effluent processing are the same as those described in Scenario AD.

### Scenarios AD-R and SP-AD-R – Recycling of the liquid fraction of the effluent from anaerobic digestion

Part of the liquid fraction of the effluent from AD is recycled and used as a diluting stream before AD, instead of water (Figure 3A and 3B). Methane production in a process that involves recirculation has not been determined, and thus the conversion factors were assumed to be the same as in the corresponding scenarios without recycling. The recycled liquid fraction contains macronutrients and trace metals, which are taken into account when calculating the amounts of these compounds to be added before AD. Based on the work of Nges et al. [34], 59, 16, 3, 13 and 5% of the total amounts of N, P, Fe, Ni, Co, respectively, are assumed to be present in the liquid phase of the AD effluent. The distribution of these compounds between the liquid fraction and the solid fraction of the AD effluent can be determined using these values and the design parameters of filter pressing of AD effluent.

### Scenarios Et-AD and Et-AD + -Ethanol process and anaerobic digestion

The slurry obtained from steam pretreatment is filter pressed (Figure 3C). The solid fraction, which contains 30% WIS, is subjected to SSF performed at 7.5% WIS and 37°C with ordinary baker’s yeast at a concentration of 3 g/L and an enzyme dosage of 20 FPU (filter-paper units)/g glucan. These conditions are the same as those applied in the underlying experiments [4] except that the yeast concentration is reduced from 5 g/L to 3 g/L based on [41]. The SSF takes place in 18 agitated non-sterile fermentors each of volume of 930 m^3^. Yeast is cultivated on part of the liquid fraction from the pretreated slurry, supplemented with molasses, while enzymes are purchased.

The ethanol concentration obtained after SSF is 2.1% by weight. This is low, and should be increased to decrease the cost of distillation. According to Wingren et al. [42] the total production cost of ethanol can be reduced by 16% by increasing the ethanol concentration of SSF broth from 2.1 to 3.1%. Pure ethanol (99.8% by weight) is produced by distillation and molecular sieve adsorption. The distillation step consists of two stripper columns and a rectifier, which are heat-integrated by operating at different pressures. Ethanol recovery is assumed to be 99.5% in each column. Wingren et al. [43] give a detailed description of the distillation system.

**Figure 4:**
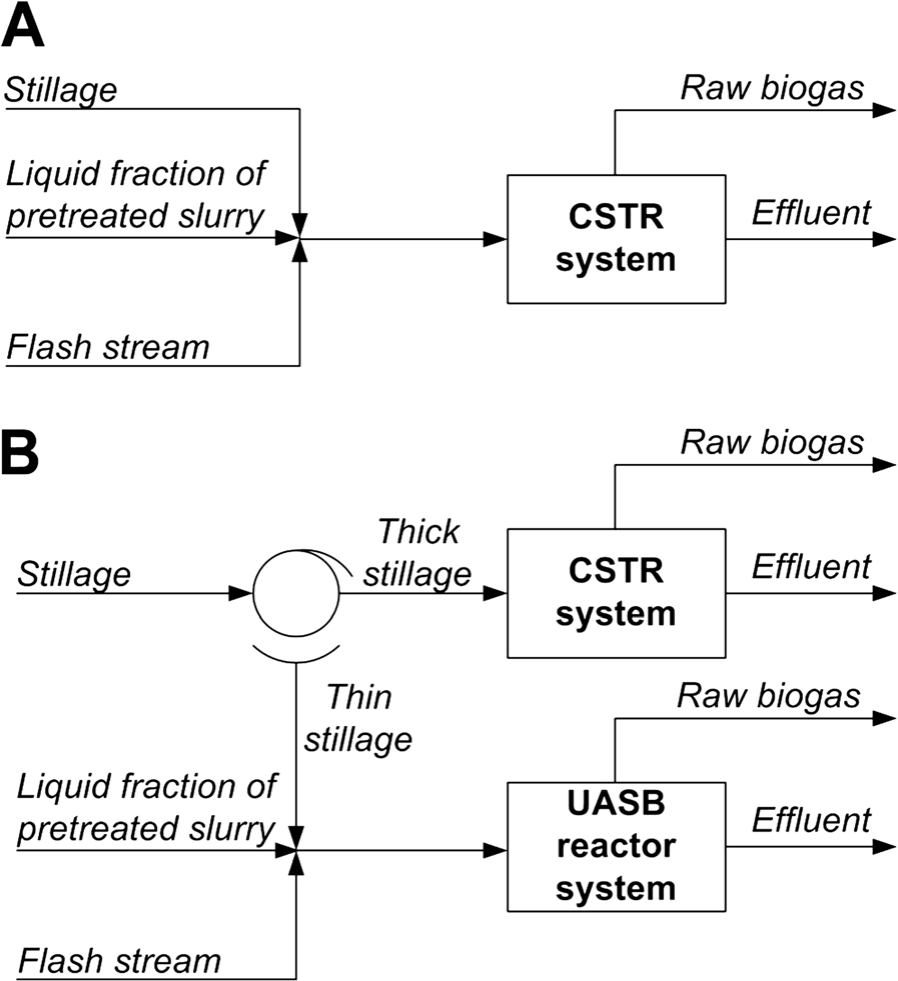
**Configurations of anaerobic digestion.** Configurations of AD after ethanol production in **(A)** Scenario Et-AD, and **(B)** Et-AD+. CSTR: continuous stirred tank reactor, UASB: upflow anaerobic sludge blanket.

The stillage from the distillation step undergoes AD, together with the liquid fraction not used for yeast propagation and the condensed flash vapours. Mixing the three streams gives a low concentration (5.9%) of DM, and two subscenarios were developed to deal with this. In Scenario Et-AD, the mixed stream is digested anaerobically in CSTRs (Figure 4A), while in Scenario Et-AD + the stillage is separated by filter pressing, and the thick stillage, together with the chopped leaves, is treated in CSTRs (Figure 4B). The separation is carried out so that the DM concentration of the effluent from the final CSTRs is 10%. The thin stillage, the condensed flash vapours and the liquid fraction of the pretreated slurry are fed to one UASB reactor with a volume of 790 m^3^, with a retention time of 3 h and upflow velocity of 5 m/h, after which the stream is passed to five parallel second-stage UASB reactors, each with a volume of 1,740 m^3^, with a retention time of 33 h and upflow velocity of 1 m/h. The organic loading rate over all reactors is 24 kg DM/(m^3^d). The design of the UASB system is based on the upflow rate from Tiwari et al. [44], and the organic loading rate from Torry-Smith et al. [45]. The AD is modelled in the same way as in Scenario SP-AD (the degradation factors of the components associated with the SSF procedure, such as enzymes, yeast, ethanol, glycerol, and succinic acid, are all assumed to be 1).

Filter pressing separates the solid and liquid fractions of the effluent from the CSTRs, while separation is not needed after the UASB reactors, since the sludge granules remain in the reactor to give a liquid effluent. This is subjected to wastewater treatment together with the liquid fraction of the CSTR effluent. The process steps of biogas upgrading and CHP (which is based on incinerating the solid fraction of the effluent from AD) are the same as those described in Scenario AD.

### Feedstock supply and cost calculations

The scenario for hemp biomass supply is based on figures obtained from cultivation in Scania, a county with an area of 1,095,000 ha, 41% of which was cultivated in 2010 [46]. The yield of hemp biomass from this region is 10.2 tonnes DM/ha [7], based on figures from cultivation trials. A 5% DM loss in the handling and storage is to be subtracted from this, to give a yield of ensiled hemp of 9.7 tonnes DM/ha. If it is assumed that the demand for biomass is 233,600 tonnes DM/year, the land area that needs to be cultivated is 24,107 ha. This calculation is based on experiments carried out with non-ensiled hemp [3,4], but the cost of ensiling must be included in the feedstock price, as fresh hemp cannot serve as feedstock throughout the year.

The actual average road transport distance is calculated from the theoretical value (based on the radius of a circle) that was derived by Overend [47], using a tortuosity factor of 1.3 [48]. The transport distance is calculated with the assumption that 5% of the agricultural land that surrounds the processing facility is used for hemp cultivation, giving an average road transport distance of 53 km. The number of transports is calculated by assuming transport in containers of volume 40 m^3^ with a density of 0.25 tonnes/m^3^, with three containers per vehicle. This gives 9.6 tonnes DM of fresh hemp per vehicle. The average speed of transport is 60 km/h, and the return trips are undertaken with an empty vehicle. The time for handling (loading three containers in the field, emptying them in the ensiling area of the bioenergy plant and subsequently unloading the empty containers in the field) is 30 minutes per transport. The hourly cost for the vehicle (truck with trailer) is 1,100 Swedish kronor (SEK)/hour (1 EUR ≈ 8.9 SEK, 1 USD ≈ 6.8 SEK). The cost for transport and handling is then 0.26 SEK/kg DM fresh hemp, which gives 0.27 SEK/kg DM ensiled hemp after ensiling losses are taken into account.

The cost of producing hemp under Swedish conditions is not available, and the model has, therefore, been based on the production cost for ensiled maize. The production cost for ensiled maize in Swedish conditions with a yield of 10 tonnes DM/ha is 1.23 SEK/kg DM, while for a yield of 12 tonnes DM/ha it is 1.04 SEK/kg DM. These costs include variable costs in the cultivation, harvest and ensiling (including labour costs), and capital costs for machinery and ensiling facilities [49]. The cost for maize with a DM yield of 10 tonnes/ha, 1.23 SEK/kg DM, has been used, increased by 10% to account for additional costs in hemp cultivation. The production cost for ensiled hemp is thus 1.35 SEK/tonne DM. The total feedstock cost becomes then 1.62 SEK/tonne DM when added transport and handling costs are included, and this figure has been used in the further calculations.

### Process design and economics

Equations for mass and energy balances were solved using the commercial flow sheeting program Aspen Plus, V7.3 (Aspen Technology, Inc., Cambridge, MA, USA). Data for the physical properties of biomass components were taken from the National Renewable Energy Laboratory database [50]. The Aspen Process Energy Analyzer V7.3 (Aspen Technology, Inc.) was used to design a near-optimal heat exchanger network and to estimate the capital cost of this network. The requirements for heating and cooling capacity were then fed back to the process model in Aspen Plus. The energy efficiency, based on the lower heating values (LHV), was defined as the energy output in the products (ethanol, biogas, electricity and district heat) divided by the energy input from feedstock (155.2 MW), molasses (7.0 MW), enzymes (9.4 MW), and the energy input in the form of electrical energy (converted to its heat equivalent, calculated using an electricity-to-heat ratio of 0.4).

The fixed capital investment cost (excluding the heat exchanger network) was estimated either with the Aspen Economic Process Analyzer V7.3 (Aspen Technology, Inc.) setting 2012 as costing year, or from vendor quotations (in the cases of the pretreatment unit, filter presses, dehydration system, CSTR anaerobic digesters with their feed systems, steam boiler, flue gas condenser, and the biogas upgrading system). Stainless steel SS304 was used as construction material in the Aspen Economic Process Analyzer, except for the UASB reactors, which were designed as carbon steel tanks. Working capital was calculated using a slight modification [43] of the recommendation of Peters et al. [51]. The annualised fixed capital cost was determined by multiplying the fixed capital investment by an annualisation factor of 0.110, corresponding to a 15-year depreciation period and an interest rate of 7%. The annualised working capital is the product of the working capital investment and the interest rate.

Table 7 summarises the cost of operation and purchase prices for materials. The cost of pH adjustment in the process has not been included, since the requirements for acid and/or base have not been determined experimentally. Further, pH calculation is not included in Aspen Plus. However, former studies [16,22] have shown that the cost of pH adjustment does not contribute to the production cost to a large extent. Other costs comprise labour, insurance, and maintenance, and have been reported in a previous study [15]. The minimum ethanol selling price (MESP) refers to the break-even point – at this price, the annual costs equal the annual incomes using the assumed market price of biogas in Table 7. Minimum biogas selling price (MBSP) is calculated similarly, however, in this case the income of ethanol is derived using the market price of ethanol in Table 7.

## Abbreviations

AD: Anaerobic digestion; CHP: Combined heat and power production; COD: Chemical oxygen demand; CSTR: Continuous stirred tank reactor; DM: Dry matter; EUR: Euro; FPU: Filter-paper unit; LHV: Lower heating value; MBSP: Minimum biogas selling price; MESP: Minimum ethanol selling price; MSEK: Million Swedish kronor; SEK: Swedish kronor; SSF: Simultaneous saccharification and fermentation; UASB: Upflow anaerobic sludge blanket; USD: United States dollar; WIS: Water-insoluble solid; YC: Yeast cultivation

## Competing interests

The authors declare that they have no competing interests.

## Authors’ contributions

ZB, EK and LB designed the study. EK provided input data for the anaerobic digestion. ZB carried out the process simulations and economic evaluation. ZB and EK analysed the results and wrote the paper. LB reviewed the manuscript. All authors read and approved the final manuscript.
